# The Relationship Between Emotional Intelligence and Conflict Management Strategies From the Nurse Managers’ Perspective

**DOI:** 10.7759/cureus.35669

**Published:** 2023-03-01

**Authors:** Manal Aseery, Sabah Mahran, Ohood Felemban

**Affiliations:** 1 Nursing, King Abdulaziz University, Jeddah, SAU; 2 Public Health/Nursing Administration, College of Nursing, King Abdulaziz University, Jeddah, SAU; 3 Public Health, King Abdulaziz University, Jeddah, SAU

**Keywords:** cultural factors, hospitals, nurse managers, conflict management strategies, emotional intelligence

## Abstract

Everyday nursing work inevitably involves conflict. Healthcare workers may experience this as a result of diverse human beliefs, knowledge, values, or emotions. In order to effectively manage and lead the nursing staff in hospitals, a multitasking leader with a wide range of abilities is needed. Multiple elements, such as the leader's personality and overall workplace conditions, might influence effective managerial leadership. Effective management leadership is affected by multiple factors, such as the personality of the leader, the general conditions in the workplace, and the quality characteristics of employees. This study aimed to investigate the relationship between emotional intelligence and conflict management strategies from the head nurses’ perspective.

Methods

The study utilized a quantitative, cross-sectional correlation design. Twenty-one hospitals in the Aseer region that are affiliated with the Saudi Ministry of Health were included in this study. A non-probability sample consisted of 210 head nurses who have at least one year of experience working as head nurses or with managerial experience. An online questionnaire involving three sections - socio-demographic, trait emotional intelligence, and conflict management - were used.

Results

The study revealed that the level of emotional intelligence was average and that the level of conflict management strategies was high. Approximately three-quarters of the sample studied were female (78.1%), and for an educational level, the majority of participants had a bachelor's degree (62.4%). Regarding the working departments, 34.3% were in general wards, while 23.3% worked in critical care. Approximately two-thirds (62%) of the sample were married; 63.8% of the participants were Saudi, and 49% had fewer than three children. Also, gender identity and emotional intelligence were significantly correlated statistically. Likewise, the level of monthly income, marital status, and nationality are significantly correlated with conflict management strategies.

Conclusions

In the current study, emotional intelligence has no statistical correlation with strategies for resolving conflicts. While the relationship between subdomains of both main variables was negative, that ruled out an important positive relationship between cooperation and well-being. Teaching nurse managers about emotional intelligence might help them more effectively handle conflicts at work. Likewise, employing emotional intelligence practices requires nurse managers to lead by example, teaching their teams how to regulate their emotions and resolve frequent workplace conflicts.

## Introduction

Conflict arises frequently in a nursing job. This might occur as a result of the many values, beliefs, and emotions that healthcare practitioners have [1]. Furthermore, hospitals with efficient nursing leadership and management require a multitasking leader who has several skills, competencies, and knowledge. According to recent theories, a variety of elements, including the leader's personality, workplace culture, and staff quality, influence how well a management team leads [2].

A study by Başoğul and Özgür found that differences in management approaches, employee views, a shortage of staff, differences in aims, and competitiveness between running groups were the most significant causes of conflicts among nurses. They made the case that inadequate conflict management can have a harmful impact on the workplace since it can result in unhealthy working conditions, unsatisfied patients, a decline in the quality of care, and higher healthcare expenditures [3]. Whether they were purposeful or not, almost all conflicts are fundamentally driven by emotions, due to the fact that conflicts arise from humans’ or companies’ beliefs about threats to their agendas. Conflict management is a crucial issue in the worldwide healthcare setting. The conflict process among nurses is significantly influenced by variations in management approaches, employee attitudes, staff shortages, inconsistencies in goals, and competitiveness between work groups [4]. Nonetheless, high-emotional intelligence managers can use this ability to comprehend and regulate their emotions in a manner that is beneficial to them as well as others. The capacity of a nurse leader to resolve interpersonal conflicts therapeutically is positively linked to emotional intelligence [5].

The aforementioned viewpoint demonstrates the fact that emotional intelligence has significantly contributed to effective leadership and has evolved into one of the most important attributes of leaders. Nurse managers who possess emotional intelligence can inspire and motivate other nurses to achieve goals that otherwise might not have been reached [5].

Originally, emotional intelligence was considered an important component of conflict resolution. However, emerging research states that emotional intelligence is a distinct concept and may predict success at work, conflict resolution, and success in life [6]. Regardless, assessing the emotional intelligence of nurse managers in Jeddah Ministry of Health (MOH) hospitals has been the target of one study in Saudi Arabia. The majority of nurse leaders, according to the study, could readily identify their emotions as they occurred, were aware of these emotions, and understood why their emotions changed. However, a smaller proportion of nursing leaders believed that they had control over their emotions [7].

Studies concerning conflict management strategies and emotional intelligence among head nurses in Saudi Arabia are lacking. This has created a knowledge gap regarding the association between these two variables. Therefore, this study came to fill the gap and aimed to investigate the relationship between emotional intelligence and conflict management strategies from the head nurses’ perspective.

## Materials and methods

Aim and design

This quantitative cross-sectional correlation study is aimed at investigating the relationship between emotional intelligence and conflict management strategies from the perspective of head nurses, to assess the level of emotional intelligence among head nurses in the Aseer Region, and to assess the conflict management strategies used by head nurses in the Aseer Region.

Setting and sample

In the Aseer region of Saudi Arabia, this investigation was conducted at 21 hospitals. All hospitals were non-profit and affiliated with the Saudi Ministry of Health. Due to a limited number of populations, the researcher involved all of the study population - 233 head nurses - in the present study; thus, a census sample was applied, in which the researcher collected and recorded the data about the members of the population to represent the entire group in all its characteristics. Male or female head nurses who have at least one year of experience working as head nurses or with managerial experience were included in the current study. While top managers and nursing supervisors were excluded from this study, a non-probability, purposive sampling technique was used.

Data collection and instruments

Two self-administered online questionnaires were used to collect data about emotional intelligence and conflict management strategies from the participants by visiting each hospital and holding a meeting with the director of nursing to introduce the researcher and explain the study's purpose. The socio-demographic data part was developed by the researcher and included gender, age, level of income per month, years of experience, marital status, number of children, nationality, department, and the level of education used to determine the personal and professional characteristics. The first tool was adopted from Petrides and used to collect information about emotional intelligence. It consists of 30 items divided between five subscales: emotionality, sociability, well-being, self-control, and global trait EI [8]. The participants were given their responses on a seven-point scale ranging from 7 (completely agree), 6 (agree), 5 (somewhat agree), 4 (neither agree nor disagree), 3 (somewhat disagree), 2 (disagree), and 1 (completely disagree). For this instrument, the overall mean was determined for all 30 items. A higher mean indicates that the head nurse has a higher level of emotional intelligence, while a lower mean indicates a lower level of emotional intelligence. The second tool was used to collect information about conflict management strategies. The tool was adopted by Martins et al. [9]. It consists of 15 items divided between five subscales: abstention, accommodation, imposition, conciliation, and collaboration. The participants were given their responses on a three-point scale ranging from 1 (rarely/never), 2 (frequently), and 3 (always). Voluntary consent was obtained from those who agreed to participate in the study. Participants’ confidentiality and anonymity were assured. The online questionnaire access link exists for a period of two months for participants’ responses, starting on August 1, 2022, until the end of September 2022. The researcher provides contact channels, electronic mail, and a phone number for inquiries related to the study.

Validity and reliability of the tools

A professional jury, comprising nursing faculty members from King Abdul-Aziz University, including assistant and associate professors, was then provided with the questionnaire. Experts with background and knowledge in the study topic review the questionnaire for content and check for face validity, consistency, and item clarification before distributing it to participants. The jury members commented that the statements in the questionnaire were appropriate, consistent, and applicable to the purpose of the study, which was to provide comments and an assessment of the questionnaire's suitability. It was determined that no adjustments were needed. To ensure that nurses who cannot speak English can comprehend the typical questionnaire, it was translated into Arabic. In order to get feedback about the questionnaire's clarity, design, arrangement, legibility, and relevance, as well as the amount of time needed to complete it, a pilot survey including 23 head nurses from the initial sample, which represented 10% of the population, has been conducted. The extent to which a questionnaire accurately represents the concept being studied determines its validity. The Cronbach's coefficient alpha is excellent (0.866) for the study domain of conflict management strategies as well as excellent (0.879) for the study domain of the Trait Emotional Intelligence Questionnaire.

Data analysis

The data were analyzed using SPSS, an IBM version 24 statistical software for the social sciences (IBM Corp., Armonk, NY). Maintaining categorical and numerical variables with demographic data, data entry for demographic variables will be done using a numerical code for each demographic variable in the questionnaire. Regarding the emotional intelligence and conflict management strategies tools, the total mean score was calculated for all items, and an independent sample t-test and a one-way ANOVA were used to investigate the association between demographic data and the total score of emotional intelligence and conflict management strategies. Additionally, Chi-square was used for conflict management strategies. To examine the link between research participants' emotional intelligence and conflict management strategies, the Pearson correlation test was used. A P-value less than 0.05 will be considered significant for an association or relationship.

Ethical considerations

In order to conduct this study, the researcher was obliged to maintain all ethical standards. King Abdulaziz University's nursing faculty, the Research Ethics Committee, provided ethical permission (Ref No. 2M. 02), and an Institutional Review Board (IRB) from the Asser Institutional Review Board at the General Directorate for Health Affairs in Aseer (REC-08-2022). Informed consent was obtained from all of the participants before completing the questionnaire. The study was anonymous; explaining the steps, main aim, and importance of the present study was done for the participants, and there was no personal risk from participating in this study. The questionnaire was kept with the researcher in an encrypted file to preserve the data's confidentiality.

## Results

Demographic data

Table 1 reveals that the participants (56.7%) were from the 30 to 39 age range, while 15.7% were in the over-40 age group. A little more than three-quarters of the sample studied (78.1%) were female, with males accounting for the remaining (21.9%). In terms of educational attainment, bachelors (62.4%) outnumbered diploma holders (31.1%). Regarding the working departments, 34.3% were general wards, while 23.3% were critical care. Approximately two-thirds (62%) of the studied sample were married, while 35.2% were single. Saudis made up the majority of participants (63.8%), while non-Saudis made up 36.2%. The majority of respondents (49%) had fewer than three children.

**Table 1:** Demographic characteristics data in the study sample (n = 210)

Demographic characteristics	N	%
Age		
25–30 years	58	27.6
30–39 years	119	56.7
˂40 years	33	15.7
Gender		
Female	164	78.1
Male	46	21.9
Years of experience		
2–4 years	42	20.0
4–7 years	40	19.0
˂7 years	128	61.0
Level of income/month		
<5000 SR	24	11.4
5000 to <10,000 SR	73	34.8
10,000 SR or more	113	53.8
Level of education		
Diploma	65	31.0
Bachelor	131	62.4
Master or more	14	6.7
The department you work in		
Critical care	49	23.3
General ward	72	34.3
Other departments	89	42.4
Marital status		
Single	74	35.2
Married	131	62.4
Widowed/divorced	5	2.4
Nationality		
Saudi	134	63.8
Non-Saudi	76	36.2
No. of children		
None	95	45.2
1–3 children	103	49.0
4 or more	12	5.7

Tables 2-3 show the level of emotional intelligence, where we have 5 with 2.4% and a low level of emotional intelligence, 172 with 81.9% and an average level, and 33 with 15.7% and a high level of emotional intelligence. While the range of results was 51 to 210, with a mean ± SD of 136,691 ± 21,074.

**Table 2:** The mean and standard deviation calculated for emotional intelligence items in the study sample (n = 210) using a one-sample T-test The mean score and p-value are significant if <0.05 using a one-sample T-test

Items	Emotional intelligence		Rank	One sample T-test (test value=3.5)		
	Mean	± SD		t	df	P-value
Emotionality						
Expressing my emotions with words is not a problem for me	5.271	± 1.577	13	16.282	209	<0.001*
I often find it difficult to see things from another person’s viewpoint.	3.671	± 1.862	23	1.334	209	0.184
Many times, I can’t figure out what emotion I'm feeling.	3.467	± 1.984	25	−0.244	209	0.808
Those close to me often complain that I don’t treat them right.	2.514	± 1.753	30	−8.147	209	<0.001*
I often find it difficult to show my affection to those close to me.	3.719	± 1.902	22	1.669	209	0.097
I’m normally able to “get into someone’s shoes” and experience their emotions.	5.343	± 1.543	11	17.312	209	<0.001*
I often pause and think about my feelings.	5.029	± 1.631	15	13.583	209	<0.001*
I find it difficult to bond well even with those close to me.	3.238	± 1.966	27	−1.930	209	0.05*
Self-control						
I usually find it difficult to regulate my emotions.	3.905	± 1.905	18	3.080	209	0.002*
I tend to change my mind frequently.	3.738	± 1.920	20	1.797	209	0.074
On the whole, I’m able to deal with stress.	5.381	± 1.521	10	17.921	209	<0.001*
I’m usually able to find ways to control my emotions when I want to.	5.295	± 1.589	12	16.372	209	<0.001*
I tend to get involved in things I later wish I could get out of.	4.424	± 1.789	16	7.481	209	<0.001*
Others admire me for being relax	5.443	± 1.506	8	18.699	209	<0.001*
Well-being						
I generally don’t find life enjoyable.	2.833	± 2.016	29	-4.793	209	<0.001*
I feel that I have a number of good qualities.	6.252	± 1.088	1	36.643	209	<0.001*
On the whole, I have a gloomy perspective on most things.	3.200	± 1.995	28	-2.179	209	0.03*
On the whole, I’m pleased with my life.	5.829	± 1.441	6	23.421	209	<0.001*
I believe I’m full of personal strengths.	5.943	± 1.217	3	29.099	209	<0.001*
I generally believe that things will work out fine in my life.	5.967	± 1.360	2	26.277	209	<0.001*
Sociability						
I can deal effectively with people.	5.838	± 1.331	5	25.448	209	<0.001*
I often find it difficult to stand up for my rights.	3.733	± 2.072	21	1.632	209	0.104
I’m usually able to influence the way other people feel.	5.171	± 1.437	14	16.851	209	<0.001*
I would describe myself as a good negotiator.	5.386	± 1.568	9	17.429	209	<0.001*
I tend to “back down” even if I know I’m right.	4.043	± 1.900	17	4.140	209	<0.001*
I don’t seem to have any power at all over other people’s feelings.	3.490	± 1.908	24	-0.072	209	0.942
General global trait EI						
On the whole, I’m a highly motivated person.	5.919	± 1.348	4	26.011	209	<0.001*
I often find it difficult to adjust my life according to the circumstances.	3.314	± 1.858	26	−1.449	209	0.149
I normally find it difficult to keep myself motivated.	3.743	± 1.947	19	1.808	209	0.072
Generally, I’m able to adapt to new environments.	5.590	± 1.475	7	20.537	209	<0.001*

**Table 3:** The level of emotional intelligence in the study sample (n = 210)

Emotional intelligence	N	%	Score	
			Range	Mean ± SD
Weak	5	2.4	51–210	136.691±21.074
Average	172	81.9		
High	33	15.7		
Total	210	100.0		

Tables 4-5 show the level of conflict management strategies, with 6 having a low level of conflict management strategies (2.9%), 80 having an average level of 38.1%, and 124 having a high level of 59% of conflict management strategies, while the range of results was 15 to 45, with a mean ± SD of 34.505 ± 5.678.

**Table 4:** The frequency and percentage of conflict management strategies employed in the study sample (n = 210)

Items		Conflict management strategies			%	Rank	Chi-square	
		Never/rarely	Frequently	Always			X^2^	P-value
Abstention								
1. Try to avoid putting yourself in an unpleasant situation and disagreements	N	48	67	95	74.13	10	15.971	<0.001*
	%	22.9%	31.9%	45.2%				
2. Avoid open discussions of differences between parties	N	60	74	76	69.21	14	2.171	0.338
	%	28.6%	35.2%	36.2%				
3. Try to keep the differences you perceive and are not yet explicit, to avoid resentment	N	51	98	61	68.25	15	17.514	<0.001*
	%	24.3%	46.7%	29.0%				
Accommodation								
4. Attempts to satisfy the expectations of the parties involved in the conflict	N	41	107	62	70.00	13	32.486	<0.001*
	%	19.5%	51.0%	29.5%				
5. Seeks to conform to the wishes of those involved	N	41	102	67	70.79	12	26.771	<0.001*
	%	19.5%	48.6%	31.9%				
6. Agrees with the solution proposed by the parties	N	31	115	64	71.90	11	51.171	<0.001*
	%	14.8%	54.8%	30.5%				
Imposition								
7. Discuss your opinion with the unit's professionals to show your view of the situation	N	16	82	112	81.90	4	68.914	<0.001*
	%	7.6%	39.0%	53.3%				
8. Firm in defending your position on the issue	N	29	73	108	79.21	6	44.771	<0.001*
	%	13.8%	34.8%	51.4%				
9. Support the solution you have in relation to the problem and usually does not give up on it	N	29	102	79	74.60	9	39.800	<0.001*
	%	13.8%	48.6%	37.6%				
Conciliation								
10. Negotiate with conflictors so that an agreement can be reached	N	23	89	98	78.57	8	47.914	<0.001*
	%	11.0%	42.4%	46.7%				
11. Adopt “give and take”, to reach an agreement	N	17	75	118	82.70	3	73.400	<0.001*
	%	8.1%	35.7%	56.2%				
12. Propose a middle ground to resolve the impasses	N	16	101	93	78.89	7	62.943	<0.001*
	%	7.6%	48.1%	44.3%				
Collaboration								
13. Seek to reflect on the issue under discussion to find an acceptable solution for you and everyone	N	14	95	101	80.48	5	67.457	<0.001*
	%	6.7%	45.2%	48.1%				
14. Exchange accurate information about the case to solve a problem together	N	11	71	128	85.24	1	97.800	<0.001*
	%	5.2%	33.8%	61.0%				
15. Try to put all concerns on the table so that issues can be resolved in the best way	N	9	81	120	84.29	2	90.600	<0.001*
	%	4.3%	38.6%	57.1%				

**Table 5:** The level of conflict management strategies in the study sample (n = 210)

Conflict management strategies	N	%	Score	
			Range	Mean ±SD
Weak	6	2.9	15-45	34.505±5.678
Average	80	38.1		
High	124	59.0		
Total	210	100.0		

Table 6 shows that there is no significant relationship between emotional intelligence and conflict management strategies, with r = −0.012 and a P-value=0.866 (greater than the significance level of 0.05).

**Table 6:** A Pearson correlation test was used to calculate the correlation between emotional intelligence and conflict management strategies

Correlations	Emotional Intelligence	
	R	P-value
Conflict management strategies	−0.012	0.866

Table 7 shows a significant negative correlation between emotionality and conciliation, where r = −0.187 and P-value = 0.007, and also a significant negative correlation between emotionality and collaboration, and a significant positive correlation between collaboration and well-being, where r = 0.193 and P-value = 0.005, but no significant correlation between other domains where all P-values are greater than 0.05.

**Table 7:** The correlation between domains of emotional intelligence (emotionality, sociability, well-being, self-control, and global trait EI) and domains of conflict management strategies (abstention, accommodation, imposition, conciliation, and collaboration

Correlations		Conflict management strategies				
Emotional intelligence		Emotionality	Sociability	Well-being	Self-control	Global trait EI
Abstention	r	−0.007	−0.039	0.086	−0.034	−0.028
	P-value	0.921	0.579	0.214	0.624	0.683
Accommodation	r	−0.015	0.085	0.065	0.119	0.060
	P-value	0.832	0.219	0.351	0.086	0.384
Imposition	r	−0.120	−0.046	0.141	0.057	0.032
	P-value	0.082	0.509	0.041	0.408	0.646
Conciliation	r	−0.187	−0.059	0.115	−0.044	−0.004
	P-value	0.007*	0.396	0.098	0.525	0.958
Collaboration	r	−0.189	−0.033	0.193	−0.004	−0.024
	P-value	0.006*	0.629	0.005*	0.956	0.732

## Discussion

Emotional intelligent

The first variable (the level of emotional intelligence in the study sample) was determined by calculating the standard deviation and the arithmetic mean of the items of emotional intelligence in the study sample. Based on the average standard deviation of the items of emotional intelligence in the study sample, the level of emotional intelligence revealed that 5 had a low level of emotional intelligence (2.4%), 172 had an average level of emotional intelligence (81.9%), and 3 had a high level of emotional intelligence (81.9%), although the score ranged from 51 to 210 with a mean ± SD (136,691 ± 21,074). This result suggests that the first variable was achieved through the results of the sample, where the largest percentage of approval of emotional intelligence items was found in the study sample, and this is consistent with [2]. The importance of emotional intelligence is highlighted as this study focused on the possibility of improving the emotional intelligence of the individual through educational intervention and training experiments. Furthermore, a study by Cleary et al. found that leaders of various healthcare services must acknowledge the vital role that these personality traits of emotional intelligence programs play in healthcare delivery since they are viewed as a crucial element of the organization's attempts to foster intercultural competency [10]. The study by Nightingale et al. [11] also supported the idea that emotional intelligence plays a crucial role in clinical communication because it helps nurses communicate with patients and their families effectively. Additionally, empowering the teams, which improves the patient experience, is a skill that emotionally intelligent managers possess. Prezerakos agreed regarding the significance of emotional intelligence. Leaders who seek to design continuing education programs and courses might need to take intercultural competence and the proper implementation of emotional intelligence into account [2].

The second variable is the relationship between emotional intelligence and demographic data (gender, years of experience, age, income level/month, department in which you work, marital status, nationality, and the number of children). With t = 2.002 and P-value = 0.048, it was discovered that there is a significant relationship between gender and emotional intelligence (increase for females than males), with mean ± SD for females of (138.037 ± 21.873) and (131.891 ± 17.304) for males. But there is no significance with other variables (years of experience, age, income level/month, department in which you work, marital status, nationality, and number of children) where the P-value increases more than 0.05 (significant level). The results of this variable also supported the findings of the study by Nightingale et al. [11], which showed how many factors, including age, culture, ethnicity, faith, and life experiences, affect a person's values, attitudes, and behaviors. It is also clearly in agreement with the study of Prufeta [12]. The results of the study showed that the average degree of emotional intelligence of female nurse leaders was on average greater than that of male nurse leaders with less than two years of experience, who had a lower level of emotional intelligence compared to other female nurse leaders, which is consistent with the current research. Also, the results of the study respected by Muhurj and Yussef [7] found that most nursing leaders can easily identify their feelings when they are experiencing them and are aware of these feelings. Leaders know why their emotions change according to the gender variable, and the Prufeta study [12], which relied on demographic data and years of experience, discovered that the emotional intelligence levels of nursing managers were average. Less experienced nurse managers score statistically lower on "usage of emotions" than those with more than two years of experience.

Conflict management

The third variable (the level of conflict management strategies in the study sample) was determined by measuring the standard deviation and the arithmetic means of the elements of conflict management strategies in the study sample. The level of conflict management strategies showed that we had 6 participants with 2.9% having a low-level conflict management strategy, 80 with 38.1% having an average level, and 124 with 59% having a high level of conflict management strategies, while the results ranged from 15 to 45 with a mean ± SD (34.505 ± 5.678). It is clear from this that the third variable was achieved through the results of the sample, where the largest percentage of approval of the items of conflict management strategies was found in the study sample, and this is consistent with the study by Basogul and Ozgür [3], where that study discovered that the conflict management strategy levels were as follows: avoidance, control, and commitment were average. Bargaining and integration were low, and integration, commitment, control, and compromise, which are strategies for managing conflict, were linked positively with emotional intelligence scores, as well as avoidance, which had a low correlation with emotional intelligence scores. Also, a study by Tuncay et al. [13] found that collaborative and bargaining strategies are the two most frequently utilized conflict resolution approaches by nurse managers.

The fourth variable is the relationship between conflict management strategies and demographic data (gender, years of experience, age, income level/month, department you work in, marital status, nationality, and the number of children). The level of monthly income and conflict management strategies were observed to be statistically significantly correlated (with the positive relationship increasing with high income), where F = 7.460 and the P-value = 0.001 less than the moral level of 0.05, with a mean ± SD for 10,000 Saudi riyals or more of 35.735 ± 5.136 and 31.458 ± 4.681 for <5000 SAR. A statistically significant relationship was observed between conflict management strategies and marital status (increase for singles), where F = 3.341 and the P-value = 0.037 is less than the moral level of 0.05 with a mean ± SD for the individual of 35.176 ± 5.927 and 28.6 ± 2.966 for widows and divorced, and so on; a significant relationship between conflict management strategies and nationality (increase for Saudis from non-Saudis) where t = 2.758 and the P-value = 0.006 is less than 0.05. But it has no significance with the other variables (gender, years of experience, age of the department in which you work, and number of children) where the P-value is more than 0.05 (significant level), and the results of this variable agree with the study by Al-Hamdan et al. [14], where it became clear that the relationship between conflict management methods and years of experience was where the type of hospital and years of expertise considerably influenced the conflict management strategies utilized.

The relationship between emotional intelligence and conflict management

**Figure 1:**
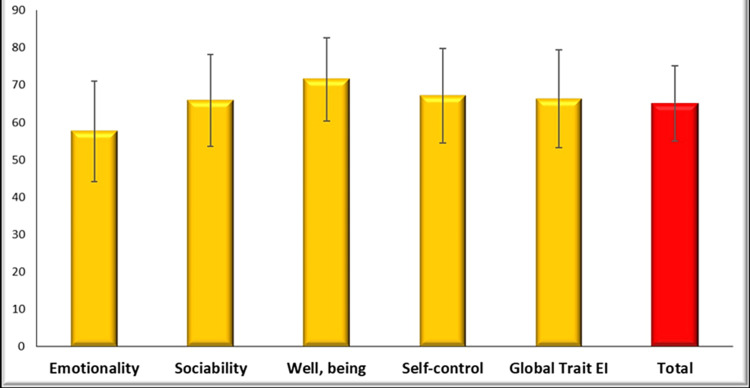
Emotional intelligence subdomain levels

**Figure 2:**
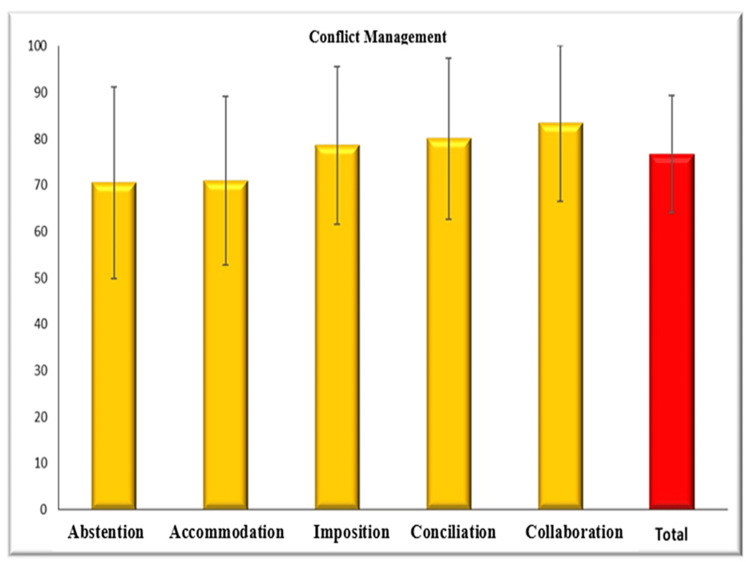
Conflict management strategies at subdomain levels

To determine how emotional intelligence and conflict management are related, Table 6 examines whether there is a positive connection between all areas, where all P-values are less than 0.05 and all correlation coefficients are positive. Table 7 also dealt with the negative moral correlation between emotionality and reconciliation, the existence of a negative moral correlation between emotionality and cooperation, and a positive moral relationship between cooperation and well-being, but there is no statistically significant correlation between other areas where the P-value increases by more than 0.05. Figure 1 shows the arithmetic average of emotional intelligence, as it was clear from this analysis that the following order was well deduced: well-being, self-control, global trait, sociability, and emotionality. Figure 2 shows this arithmetic means of conflict management, as it became clear from this analysis that the following order has been deduced: collaboration, conciliation, imposition, accommodation, and abstention.

Study limitations

The difficulty of distributing questionnaires to the selected study sample, consisting of 21 hospitals, is due to the geographical difficulty of reaching these hospitals, as they are located in distant and remote places. In addition, the hard way to some of the included hospitals in the study, as it is known about the mountain nature of the region. Respondents may give responses that are socially desirable rather than ones that are honest about the items on an instrument. Due to the study's focus on one level of managers, the results cannot be generalized.

Recommendations

Based on the research findings, which investigate the relationship between emotional intelligence and conflict management strategies from the nurse manager's perspective, it is recommended that the findings of the study be used to inform and improve clinical nursing practice in the following ways: emphasize the importance of emotional intelligence training for nurse managers and other healthcare leaders; develop specific conflict management strategies that are tailored to the unique needs of the nursing profession; incorporate emotional intelligence assessments as part of the recruitment and selection process for nurse managers; create opportunities for continuing education and professional development for nurse managers to improve their emotional intelligence and conflict management skills; encourage an organizational culture that values emotional intelligence and effective conflict management; develop mentoring and coaching programs that promote the use of emotional intelligence and effective conflict management strategies among nurses and nurse managers.

## Conclusions

The research is designed to reach a set of important results: the capacity to assess nursing managers' emotional intelligence level, examine the conflict management strategies employed by head nurses, and recognize the relationship between emotional intelligence and conflict management strategies from the head nurses' perspective. The results illustrate that there is a strong positive correlation among all subdomains of emotional intelligence. There is a significant relationship between gender and emotional intelligence. In addition, the results prove the existence of a significant relationship between all subdomains of conflict management strategies and a significant relationship between the level of income per month, marital status, nationality, and conflict management strategies.
